# Clinics at the Buffalo General Hospital by Professor Rochester

**Published:** 1881-12

**Authors:** C. C. Fredericks


### Clinical Reports.

### Clinics at the Buffalo General Hospital by Professor Rochester.

Reported by Dr. C. C. Fredericks.

*Case 1.* E. P., æt. 34, married, and by occupation an iron moulder, entered the hospital Aug. 30, 1881. Patient said that he had been sick for six months, beginning with intermittent fever, which was persistent, and even since recovery from it, had had great pain over the region of the liver and stomach. Appetite was poor, was constipated, had failed rapidly in strength and was considerably emaciated. Examination showed the liver to be enlarged, the area of dullness extending from the nipple to about two inches above the crest of the ilium. He had always been temperate. Pressure over the liver produced pain. Heart and lungs were found to be normal, and an examination of the urine detected nothing unusual. The evacuations from the bowels, although constipated, were otherwise natural. The pulse rose from 80 to 100, and at night the temperature ran from 100° to 102°; no chills during either day or night. Patient was given tonic treatment, rest and counter-irritation, with warm applications over the affected side, and a mild laxative daily. He remained about the same till Sept. 15, when he began to have irregular and severe chills associated with excruciating pain just beneath the ribs. This pain was so severe that large doses of morphine by the mouth (gr. ½–1) would not control it. Morphine (gr. ½) hypodermically, would give relief for a short time. The temperature then ranged from 102° to 105° at night. On Sept. 20, chills were less frequent, and on Sept. 25 the patient was improving. By Oct. 1 he was again sitting up, ate quite well, had no more chills, temperature was only slightly above normal; still he had a dull heavy pain under the ribs on right side. On Oct. 15, chills and great pain again returned. Examination of the chest showed the area of dullness to extend about two inches above the nipple. Examination from time to time showed this area extending still higher. Effusion into the plura being suspected, a hypodermic needle was thrust through the chest wall posteriorly, reaching no fluid. This was repeated several times, with the same result. The case was diagnosed as hepatic abscess, but as no fluctuation could be determined, it was thought to be multiple abscess. Oct. 19, Dr. Rochester saw the patient at his clinic, and after careful examination thought the case abscess of the liver, but could not determine the existence of a distinct pus cavity. The case grew worse, with anasarca of the lower extremities, followed by that of the whole body. Nov. 9th the patient died. The autopsy was made the same day before the medical class.

The attention of the students was especially directed to the position of the abscess, occupying as it did the upper part of the liver and extending far upwards into the cavity usually filled by lung tissue. In this respect especially was the case rare and interesting. The causes of such abscesses were also considered at some length, being due to a local inflammation of obscure nature, to the presence of stones in the ducts of the liver or to echinococci.

On laying open the chest and abdomen, the liver was found to extend from the second rib to the crest of the ilium, thus occupying nearly all the right side of the trunk. Its outer surface at points was covered with inflammatory lymph. The diaphragm had been pushed before the ascending liver, thus nearly obliterating the right chest and lung. The whole liver was hypertrophied, but the greatest enlargement was from the superior curved surface of the right lobe, in the form of an abcess, which stood like a dome, its base about eight inches broad, and arching to the lower border of the second rib. The whole organ when removed weighed 16¾ lbs. The abcess was punctured and evacuated, and the liver then weighed 6¼ lbs., making the weight of the contained pus 10½ lbs. No other abcesses were found, hence the reason why fluctuation could not be detected, the abcess being entirely beneath the ribs. No other organs were found diseased. Dr. Rochester said that this man might have been relieved temporarily, had the exact location of this abcess been determined and its contents evacuated, but he thought nothing would have given him any chance of restoration to health.

*Case 2.* G. R., æt. 50, single, a laborer, entered the hospital Nov. 19th, 1881, very feeble. Gave a meagre history, saying he had been sick only two weeks, and had great pain in his right side. Coughed constantly, raising large quantities of purulent matter; pulse was feeble, 120 to 140 per minute; temperature, 102° to 104°. On examination, the heart's apex was found between the sixth and seventh ribs to the left of a line perpendicular through the nipple. Percussion over the right chest gave dullness in the upper part, flatness below, dullness over the whole left chest. Auscultation gave mucous rales over left lung and upon lobe of right, and very weak respiratory murmur, if any, over lower lobes of right. The patient was moribund and died the next evening. The autopsy was held before the medical class, Nov. 23d. On opening the chest, the right side was found to contain about two quarts of purulent fluid, the lower lobe of the right lung was hepatized and bound to the costal and diaphragmatic pleura by a thick, false membrane of recent formation. Throughout both lungs, there was an abundant deposit of tubercle undergoing softening in spots, a cavity the size of a small egg being found in the middle of the right lobe, and several smaller ones in the upper lobe.

This then was a case of tuberculosis, pluro-pneumonia and empyema. Dr. Rochester thought the tuberculosis the primary, and the others only secondary, remarking that any case of tuberculosis is liable to such complications, and that they would occur more frequently were it not for the conservatism shown by nature in causing the opposing pleural surfaces to unite opposite to a cavity, and thus preventing the rupture of the cavity into the plura and the consequent pneumo-hydrothorax succeeding to such conditions.

